# TOPK inhibits autophagy by phosphorylating ULK1 and promotes glioma resistance to TMZ

**DOI:** 10.1038/s41419-019-1805-9

**Published:** 2019-08-05

**Authors:** Hui Lu, Juanjuan Xiao, Changshu Ke, Xiaofang Ni, Ruijuan Xiu, Qin Tian, Huaxiong Pan, Ling Zou, Fei Wang, Tengfei Ma, Xinying Ji, Ping Yuan, Lin Liu, Jianmin Zhang, Wei Jia, Qiuhong Duan, Feng Zhu

**Affiliations:** 1 Department of Biochemistry and Molecular Biology, School of Basic Medicine, Huazhong University of Science and Technology, 430030 Wuhan, Hubei PR China; 2 Department of Pathology, Tongji Hospital, Tongji Medical College, Huazhong University of Science and Technology, 430030 Wuhan, Hubei PR China; 3 Department of Pathology, Union Hospital, Huazhong University of Science and Technology, 430030 Wuhan, Hubei PR China; 4 Henan International Joint Laboratory of Nuclear Protein Regulation, Henan University Medical Center (HUMC), 475004 Kaifeng, Henan PR China

**Keywords:** CNS cancer, Autophagy

## Abstract

ULK1, the upper-most protein of the ULK1 complex, is emerging as a crucial node in autophagy induction. However, the regulation of ULK1 is not fully understood. In this study, we identified TOPK (T-LAK cell-originated protein kinase), an oncokinase, as a novel upstream kinase to phosphorylate ULK1. We found that TOPK could directly bind with and phosphorylate ULK1 at Ser469, Ser495, and Ser533. The phosphorylation of ULK1 at Ser469, Ser495, and Ser533 by TOPK decreased the activity and stability of ULK1. In addition, we want to examine the initiation of autophagy because the reduction activity of ULK1 reduces the occurrence of autophagy. We demonstrated that TOPK could inhibit the initiation and progression of autophagy in glioma cells. Furthermore, TOPK inhibition increased the sensitivity of glioma cells to temozolomide (TMZ). This discovery provides insight into the problem of TMZ-resistance in GBM treatment.

## Introduction

Glioblastoma multiforme (GBM) accounts for more than 50% of malignant gliomas. The median survival time of GBM is 15 months because of being resistant to the therapy^1^. Temozolomide (TMZ) is the first-line chemotherapeutic drug of GBM. However, only 45% of the patients were effective, most of the patients were resistant to TMZ in the course of therapy^2^. The mechanism of glioma resistance to TMZ is complex, including DDR^3^, EGFR overexpression^4^, p53 mutations^5^, and autophagy activity changes^6^. The role of autophagy in TMZ resistance is unclear. Liu T. et al. found that Momelotinib induced autophagy and increased the sensitivity of glioma cells to TMZ^7^. On the other hand, Zou Y. et al. found that TRPC5 induced autophagy, but promoted the resistance of glioma cells to TMZ. Therefore, it is crucial to elucidate the role of autophagy in TMZ resistance.

Macroautophagy (hereafter referred to “autophagy”) is a mechanism by which cells degrade damaged organelles or proteins through lysosomal pathways^8^. The process includes initiation, nucleation, elongation, the fusion of autophagosomes with lysosomes, and degradation of contents^9^. Studies show that abnormal autophagy is closely related to the development of many diseases, such as neurodegenerative diseases^10^, inflammatory bowel disease^11^, pulmonary arterial hypertension^12^, diabetes^13^, and tumors^14^. Autophagy plays an important role in the treatment of tumors. But the regulatory mechanism is complex. Therefore, understanding the role of autophagy provides a new window for designing treatment strategies for tumors.

The ULK1 complex, including ULK1, ATG13, ATG101, and FIP200^15^ is generally considered to be the initiator of autophagy^16–18^. As a core member of the ULK1 complex, ULK1 is a crucial node to initiate autophagy, but the regulation of ULK1 has not been fully understood. Studies show that ULK1 can be ubiquitinated^19^, acetylated^20^, and phosphorylated^21^, the research is mainly focused on its phosphorylation. At present, only three direct upstream kinases have been reported to phosphorylate ULK1, including AMPK, mTOR, and p38 MAPK. AMPK can activate ULK1 by phosphorylating ULK1 at Ser317, Ser777 to enhance the initiation of autophagy^21,22^. mTOR directly phosphorylates ULK1 at S757 to prevent ULK1 activation^19,23^. p38 MAPK decreases the activity of ULK1 by phosphorylating ULK1 at Ser504 and Ser757, and inhibiting autophagy^24^. However, whether other sites of ULK1 can be phosphorylated by other kinases and its effect on autophagy after phosphorylation is not well understood, so the regulation of ULK1 needs further study.

Many protein kinases are activated during drug resistance^25^. However, whether these tumor-associated kinases (oncokinases) are involved in the regulation of ULK1, thereby affecting drug resistance in tumor, the current research is not very clear. T-LAK cell initiation protein kinase (TOPK) participates in cell cycle regulation^26^, and promotes tumor cell growth, proliferation, migration, and invasion. TOPK is overexpressed in lots of malignant tumors, including colon cancer, breast cancer, glioma, and so on^27–29^. Here, we identified TOPK as a novel upstream kinase to phosphorylate ULK1. TOPK could directly bind with and phosphorylate ULK1 at Ser469, Ser495, and Ser533. The phosphorylation of ULK1 by TOPK decreased its activity and stability. Moreover, TOPK could inhibit the initiation of autophagy in glioma cells. Furthermore, TOPK inhibition increased the sensitivity of glioma cells to TMZ. This discovery provides insight into the problem of TMZ-resistance in GBM treatment.

## Materials and methods

### Cell lines and cell culture

H4, Hs683, U118MG, A172, and HEK293T cells were purchased from American Type Culture Collection (ATCC), and cultured following their culture procedures. The cells were cultured with Dulbecco’s minimum essential medium (DMEM) containing 10% fetal bovine serum (FBS), and cultured at 37 °C and 5% CO_2_. Chloroquine (CQ) diphosphate salt (catalog: C6628) and TMZ (catalog: 76899) were purchased from Sigma-Aldrich (St. Louis, MO, USA). Rapamycin (catalog: HY-10219), Wortmannin (catalog: HY-10197), OTS964 (catalog: HY-12467), and Cycloheximide (CHX, Synonyms: Garamycin A; Actidione) (catalog: HY-12320) were bought from MedChem Express (Monmouth Junction, USA).

### Clinical specimens

The human gliomas (WHO Grade I–IV) collection consisted of surgically resected gliomas specimens from nine patients. The clinical specimens were obtained from the Pathology Department of Tongji Hospital and approved for research by the Research Ethics Committee of Tongji Medical College, Huazhong University of Science and Technology.

### Knockdown of TOPK

TRC lentiviral shRNA constructs were designed to knockdown TOPK. They were: 1. 5′-GGGAACTAGGCCACCTATTAA-3′; 2. 5′-GAAGTGTGGCTTGCGTAAATA-3′; 3. 5′-GTAATGATCATTATCGAAGTG-3′; 4. 5′-GCCTTCATCATCCAAACATTG-3′; 5. 5′-GATTCCACACATTAATCTTTC-3′. A mock shRNA whose sequence was 5′-CCTAAGGTTAAGTCGCCCTCG-3′ was used as a negative control.

### Western blot

The cells (3 × 10^6^) were cultured in a culture dish with a diameter of 10 cm at ~80% confluence. After different treatment, 300 μl of RIPA buffer (150 mM NaCl, 50 mM NaF, 1 mM Na_3_VO_4_·12H_2_O, 5 mM sodium pyrophosphate, 1% deoxysodium cholate, 1% NP-40, 1 mM EDTA, 50 mM Tris-HCl and 1 mM phenylmethylsulfonylfluoride) was added to disrupt cells. The samples were collected and ultrasonicated for three times, 15 s each time, and then centrifuged for 10 min at 12,000 rpm. The content of protein was quantified through the Bradford method. 5 × SDS loading buffer was added to the samples. Then, the samples were heated with a protein heater at 95 °C for 10 min. Later, the samples were resolved by 6–15% of SDS–PAGE and transferred onto the PVDF membrane (Millipore). After that, the membrane was blocked with 5% of fat-free milk or BSA for 20–30 min and washed three times with TBST, 5 min each time. Then the membrane was incubated with the indicated primary antibodies overnight at 4 °C. The membrane was then washed three times with TBST, 5 min each time and incubated with HRP-conjugated secondary antibody (EarthOx) for 1 h at room temperature. Chemiluminescence method (BIORAD) was used to detect the signals. The following primary antibodies were used: rabbit anti-P62/SQSTM1 (1:1000, Proteintech Group, 18420-1-AP), mouse anti-TOPK (1:1000, Santa Cruz, sc-293028), rabbit anti-LC3B (1:1000, Cell Signaling Technology, 2775), rabbit anti-Caspase-3 (1:1000, Cell Signaling Technology, #9662), rabbit anti-Cleaved-Caspase-3 (1:500, Cell Signaling Technology, #9664), rabbit anti-ULK1 (1:500, Cell Signaling Technology, 8054), rabbit anti-ATG13 (1:1000, Cell Signaling Technology, 13468), rabbit anti-phospho-ATG13 (1:500, Cell Signaling Technology, 26839), rabbit anti-Histone H3 (1:1000, Cell Signaling Technology, 4499), rabbit anti-phospho-Histone H3 (1:1000, Cell Signaling Technology, 3377S), rabbit anti-HA (1:1000, Cell Signaling Technology, 3724), mouse anti-HA (1:200, Pregene, PRE003F), mouse anti-Alpha Tubulin (1:1000, Proteintech Group, 66031-1-Ig), mouse anti-His (1:1000, Pregene, PRE004F), rabbit anti-Flag (1:1000, Sigma-Aldrich, F7425). Antibody-bound proteins were visualized using a chemiluminescence detection kit (BIO-RAD, USA). Luminescent image analyzer was ChemiDoc XRS+ with Image Lab Software (BIO-RAD, USA). Protein levels were compared by measuring mean density values. The band densities were determined by Image J.

### MTT assay

H4 and Hs683 cells (1 × 10^4^/well) were seeded in 96-well plates for 24 h. Then fresh media with different concentrations of OTS964 was added for 24 and 48 h, respectively. After that, 90 μl of fresh media with 10 μl of MTT (0.5 mg/ml) was added per well for 4 h, then the culture media was discarded, and 150 μl of DMSO was added. The absorbance of each pore was measured at OD490 nm by enzyme-linked immunosorbent assay (Elisa) when the crystal was fully dissolved. Four multiple holes were set up in all MTT experiments, and the average absorbance values were calculated. The non-treated cells were as negative control.

### Electron microscopy

H4-shRNA cells (2 × 10^6^) were seeded onto the coverslips for 24 h, and then washed with 1 × PBS for three times before being treated with HBSS for 6 h and CQ (50 μM) for 3 h, after that, cells were digested with trypsin and collected into 15 ml centrifuge tube with 1 × PBS, then centrifuged for 5 min at 800 rpm. The concentrated cell pellet was then transferred into the 1.5 ml EP tube, and centrifuged for 10 min at 1500 rpm. After that, the supernatant was discarded, glutaraldehyde fixative was slowly added along the inner wall to avoid dispersing cells and being fixed for 2 h. Samples were photographed by transmission electron microscopy. The numbers of autophagosomes were analyzed using Prism 5 software. The data were presented in the form of mean ± SD, *P < 0.05, **P < 0.01, ***P < 0.001.

### GFP-LC3 fluorescence microscopy

H4-shRNA cells were seeded onto the coverslips for 24 h, and then transiently transfected with GFP-LC3 for 48 h. After that, the cells were treated with HBSS for 6 h and CQ (50 μM) for 3 h, and then cells were washed with 1 × PBS for three times, fixed with 4% paraformaldehyde and ruptured with 0.5% Triton-PBS for 20 min. Samples were photographed by fluorescence microscope. The average number of autophagosome per cell (nine cells) were quantified manually and analyzed using Prism 5 software. The data were presented in the form of mean ± SD, *P < 0.05, **P < 0.01, ***P < 0.001.

### Softagar assay

The different cell lines (8 × 10^3^/well) were in a six-well plate and cultured in 1 ml of 0.33% BME (Eagle-based medium, Sigma-Aldrich Corp.) agar (Sigma-Aldrich) containing 10% FBS over 3 ml of 0.5% BME agar with 10% FBS. The cells were incubated for 7–14 days at 37 °C and 5% CO_2_, and the clones were observed under the microscope. The numbers of clones were analyzed using Prism 5 software. The data were presented in the form of mean ± SD, *P < 0.05, **P < 0.01, ***P < 0.001.

### Flow cytometry

Cells (2 × 10^5^/well) were seeded in a six-well plate and cultured at 37 °C and 5% CO_2_ for 24 h. Then cells were induced apoptosis according to the test protocol, digested with trypsin, and centrifuged at 300 × *g* for 5 min. The supernatant was discarded, and the cells were collected, washed with PBS, the supernatant was discarded. The cells were resuspended with 500 μl of diluted 1 × Annexin V-binding buffer working solution and added 5 μl of Annexin V-FITC and 5 μl of propidium iodide (PI) staining solution. The cell suspension was mixed gently and blocked at room temperature for 15–20 min, then checked using the FACS Diva machine immediately. The percentage of apoptotic cells were counted automatically using Flowjo software and analyzed using Prism 5 software. The data were presented in the form of mean ± SD, *P < 0.05, **P < 0.01, ***P < 0.001.

### Ni-NTA His-TOPK purification

PET46-His-TOPK were expressed in *E. coli* BL21 bacteria. Bacteria grew at 37 °C to an absorbance of 0.6–0.8 at 600 nm. After that, 1 mM isopropyl β-d-thiogalactopyranoside (IPTG) was added for 3 h to induce protein high expression. The bacteria were centrifuged at 3000 rpm for 10 min and then washed with cold 1 × PBS for three times. The bacteria precipitates were frozen at −80 °C and thawed at 37 °C for three times, respectively. The bacteria precipitates were sonicated for 20 min after being added cold 1 × PBS and then centrifuged for 10 min at 12,000 rpm. The supernatant was collected and purified with nickel–nitrilotriacetic acid agarose (Qiagen) overnight at 4 °C and then washed with 20 or 40 mM imidazole. After that, the samples were resolved by 10% SDS–PAGE and visualized by Coomassie brilliant blue staining.

### Ni-NTA His-ULK1-FL/KD/SPR/CTD purification

293T cells (1 × 10^4^/dish) were seeded in 10 cm dishes for 24 h, then transfected with pcDNA4-His-ULK1-FL/KD/SPR/CTD or its empty vector. 48 h later, cells were lysed with 600 μl of His-tag purification buffer (50 mM NaH_2_PO_4_, 50 mM NaF, 250 mM NaCl, 0.5% NP-40, and 1 mM phenylmethylsulfonylfluoride) plus 10 mM imidazole. The lysate was transferred into a 1.5-ml microfuge tube and saved in −20 °C overnight. Then, the cell lysate was thawed at 37 °C for 30 min and centrifuged at 10,000 × *g* for 10 min. The supernatant was transferred into a new 1.5-ml microfuge tube and mixed with 50 μl of 50% slurry of Ni-NTA beads (Qiagen). The mixtures were rotated at 4 °C for 24 h. The beads were washed four times each with 250 μl of wash buffer (50 mM NaH_2_PO_4_, 50 mM NaF, 300 mM NaCl, 0.05% Tween-20, pH 8.0) plus 20 mM imidazole by centrifugation at 1000×*g* for 2 min. The bound proteins were stored at 4 °C.

For mass spectrometry (MS) assay, Ni-NTA His-ULK1-FL was eluted out with 50 μl of 100 mM imidazole in wash buffer by centrifugation at 1000 × *g* for 2 min, stored at −20 °C. It is worth noting that there is no degreaser in buffers for MS.

### Immunoprecipitation and pull down assay

Cells in 10 cm cell culture plate were harvested at ~80% confluence, and disrupted with 500 μl of IP buffer (150 mM NaCl, 1 mM EDTA, 1 mM DTT, 1% NP-40, and 50 mM Tris–HCl, pH 7.4) and repeated passage through a 21-gauge needle. Then cells were centrifuged at 12,000 rpm for 10 min. The supernatant was collected and incubated with 1.0 µg of the control IgG together with 20 µl of Protein A/G-Agarose overnight at 4 °C, then centrifuged at 3000 rpm for 3 min. The supernatant was transferred into the 1.5 ml EP tube and incubated with mouse monoclonal anti-HA antibody together with 20 µl of Protein A/G-Agarose overnight at 4 °C, then centrifuged at 3000 rpm for 3 min. The supernatant was carefully aspirated and discarded, and immunoprecipitates were collected. After that, the samples were resolved by 10% SDS–PAGE and analyzed using western blot. The same amount of protein 1–2 mg was used for pull-down assay.

### In vitro kinase assay

The TOPK active kinase (2 μg) and the ULK1 peptides (10 μg) in a 30 μl reaction containing 1 μCi [γ-^32^P] ATP were incubated at 37 °C for 2 h. After that, 5 × SDS loading buffer was added to samples, and then the samples were resolved by SDS–PAGE gel without water and analyzed by autoradiography.

For MS assay, TOPK (2 μg) served as an active kinase and His-ULK1-FL (10 μg) protein as a substrate. They were incubated with 1 mM ATP at 37 °C for 2 h.

### MS assay

As for solution samples, 8 M urea/100 mM Tris–HCl solution (pH 8.0) was added 1:1 to the protein for protein denaturation. The protein was centrifuged at 12,000 × *g* for 15 min. The supernatant was transferred, and 10 mM (final concentration) dithiothreitol (DTT) was added. Then it was incubated at 37 °C for 1 h for a reduction reaction to open the disulfide bond. Subsequently, to block the sulfhydryl group, iodoacetamide (IAA) was added at a final concentration of 40 mM for an alkylation reaction at room temperature and in the dark. 100 mM Tris–HCl solution (pH 8.0) was added to dilute Urea to a concentration <2 M. Then 1 μg trypsin was added, and it was incubated at 37 °C for enzyme digestion. The next day, IAA was added to terminate the enzyme digestion, then it was centrifuged at 12,000 × *g* for 15 min. The supernatant was transferred and desalinated using Sep-Pak C18. The phosphorylated peptides were enriched using IMAC approach and dried with a centrifugal concentrator. They were stored at −20 °C for machine test.

MS was performed using SCIEX’s TripleTOF 5600 liquid-MS system. The peptide samples were inhaled by an automatic sampler and combined to a C18 capture column (5 m, 5 × 0.3 mm), and then eluted to an analytical column (75 μm × 150 mm, 3 μm particle size, 100 pore size, Eksigent). Two mobile phases (mobile phase A: 3% DMSO, 97% H_2_O, mobile phase B: 3% DMSO, 97% ACN, 0.1% formic acid) were used to establish a 30-min analytical gradient (0 min in 5% B, 15 min of 5–35% B). 1 min of 35–80% B, 80% B for 5 min, 0.1 min of 80–5% B, 5% B for 8.9 min). The liquid flow rate was set to 300 nL/min. In mass spectrometric IDA mode analysis, each scan cycle included a full MS scan (*m*/*z* range of 350–1500, ion accumulation time 250 ms), followed by 40 MS/MS scans (*m*/*z* range of 100–1500, ion accumulation time 50 ms). The condition of MS/MS acquisition was that the charge number of the master ion signal was 2–5 when the signal was more than 120 cps. The dynamic exclusion time of repeated ion acquisition was set to 18 s.

The MS data generated by TripleTOF 5600 were retrieved via Protein Pilot (V4.5), Paragon was the adopted database retrieval algorithm. The database used for retrieval was the human proteome reference database in Uniprot. The retrieval parameters were as follows: selected Identification as Sample Type; IAA as Cys Alkylation; Trypsin as Digestion; Phosphorylation emphasis as Special Factors; Search Effort was set as Rapid ID. The search results were screened under the standard of Unused (>1.3). The entry and polluted proteins were deleted. Remaining information of identification was for later analysis.

### Statistics

The experiments were repeated at least three times, and all data represent the results of triplicate experiments. The data were presented in the form of mean ± SD, *P < 0.05, **P < 0.01, ***P < 0.001. Statistical analysis charts were made with Prism 5, and *t*-test was used in the statistical analysis.

## Results

### TOPK inhibits autophagy in glioma cells

**Fig. 1:**
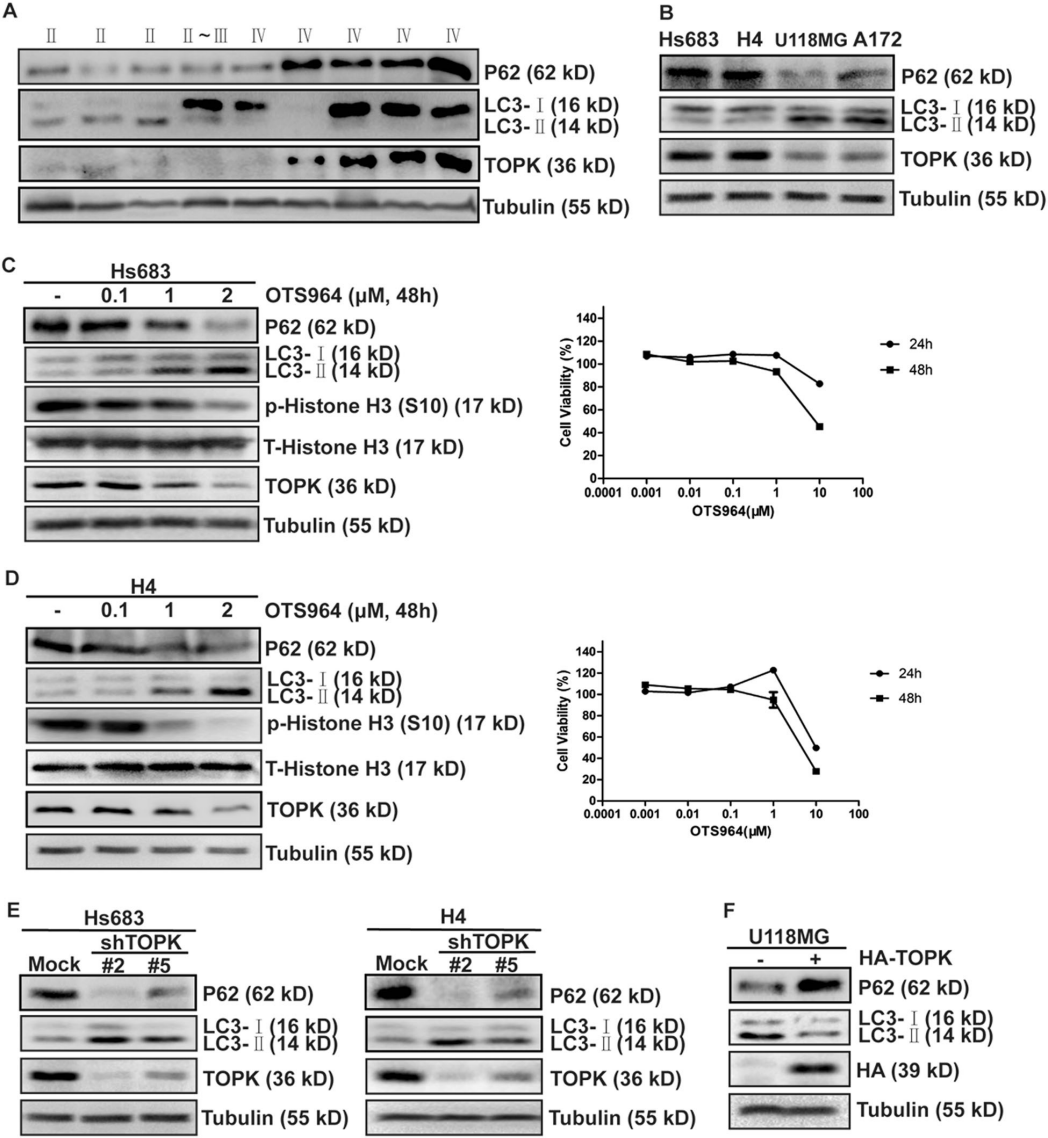
TOPK Inhibits autophagy in glioma cells. **a** The expression of TOPK, LC3-II, and P62 in clinical specimens. P62, LC3B, TOPK, and Tubulin were analyzed using western blots. **b** The expression of TOPK, LC3-II, and P62 in four glioma cells. P62, LC3B, TOPK, and Tubulin were analyzed using western blots. **c** Differing concentrations of OTS964 were used to treat Hs683 cells for 24 and 48 h, respectively. Cytotoxicity was measured by MTT assay (right). Hs683 cells were treated with different concentrations of OTS964 for 48 h then the whole cell lysates were analyzed by western blots using indicated antibodies (left). **d** Differing concentrations of OTS964 were used to treat H4 cells for 24 h and 48 h, respectively. Cytotoxicity was measured by MTT assay (right). H4 cells were treated with different concentrations of OTS964 for 48 h, then the whole cell lysates were analyzed by western blots using indicated antibodies (left). **e** TOPK knockdown in Hs683 (left) and H4 (right) cells enhanced the expression of LC3-II and decreased the expression of P62. TOPK-silencing-Hs683 and TOPK-silencing-H4 cells were harvested at 80% confluence, and resolved by SDS–PAGE and analyzed using western blots. Data represent the results of triplicate experiments. **f** TOPK overexpression in U118MG reduced the expression of LC3-II and increased the expression of P62. pcDNA3 and pcDNA3-HA-TOPK were stably transfected into U118MG cells as indicated. P62, LC3B, HA-TOPK, and Tubulin were analyzed using western blots

Recent reports show that TOPK is a biomarker^30^. Also, high-grade glioma shows low expression of LC3-II^31^. To assess the relationship between TOPK and autophagy, we detected the expression of TOPK and markers of autophagy^18,32^, LC3-II, and P62 in different clinical specimens. The results showed that high-grade glioma exhibited relatively high expressions of TOPK and P62 and a low expression of LC3-II (Fig. 1a). Also, we analyzed the expression of TOPK, LC3-II, and P62 in four human glioma cell lines and found that cells with relatively high expression of TOPK expressed high P62 and low LC3-II (Fig. 1b). These findings suggested that TOPK might be related to autophagy. To further clarify whether TOPK regulates autophagy, different concentrations of OTS964, a specific TOPK inhibitor, were added to Hs683 and H4 cells, and LC3-II and P62 were detected. The results showed that OTS964 treatment increased the expression of LC3-II and decreased the expression of P62, and both were in a dose-dependent manner (Fig. 1c, d). Secondly, shRNA was used to silence TOPK in Hs683 and H4 cells to establish TOPK-silencing stable cell lines, and LC3-II and P62 were detected. The results showed that compared with the non-silencing group, expressions of LC3-II in TOPK-silencing groups significantly increased, and expressions of P62 significantly decreased (Fig. 1e), indicating that silencing TOPK promoted the expression of LC3-II and inhibited the expression of P62. Finally, overexpressing HA-TOPK in U118MG cells to establish TOPK overexpressing stable cell lines, and LC3-II and P62 were detected. The results showed that overexpressing TOPK decreased the expression of LC3-II, increased the expression of P62 (Fig. 1f). Based on the results, we conclude that TOPK inhibits autophagy in glioma cells.

**Fig. 2:**
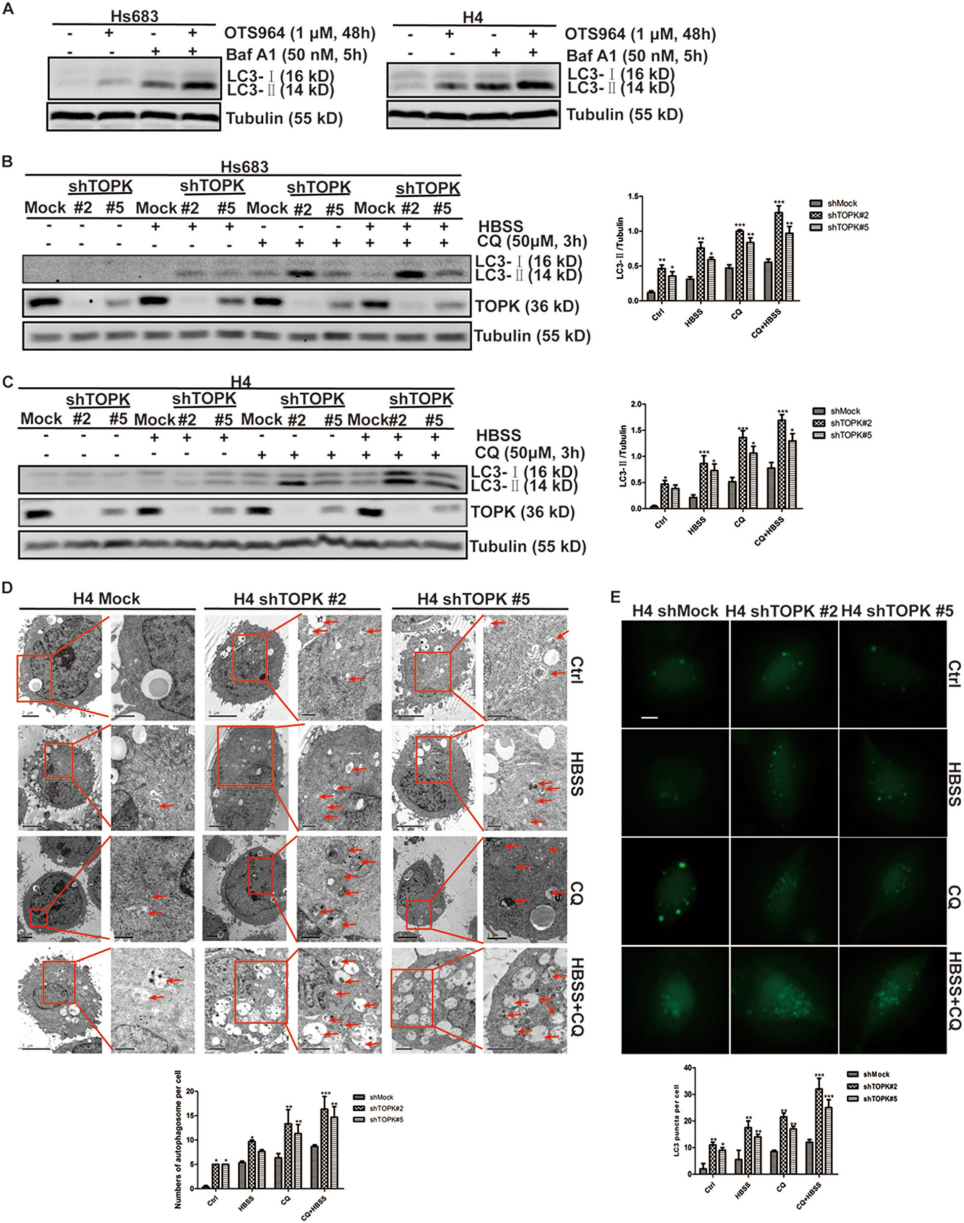
TOPK inhibits autophagy initiation in glioma cells. **a** Hs683 (left) and H4 (right) cells were treated with OTS964 for 48 h after seeding for 24 h. Baf A1 (50 nM) was added 5 h before the cells were harvested. Samples of whole cell lysates were analyzed by western blots using the indicated antibodies. TOPK-silencing-Hs683 **b** cells and TOPK-silencing-H4 **c** were treated with HBSS for 6 h after seeding for 24 h. CQ (50 μM) was added 3 h before the cells were harvested. Samples of whole cell lysates were analyzed by western blots using the indicated antibodies. (Fig. 2**b** and **c**, left). Data of **b** and **c** represent the results of triplicate experiments. The densities of LC3-II/Tubulin in Hs683 (Fig. 2**b**, right) and H4 (Fig. 2**c**, right) were determined by Image J. The data were presented in the form of mean ± SD, *P < 0.05, **P < 0.01, ***P < 0.001. **d** H4-shRNA cells were seeded onto the coverslips for 24 h, and then treated with HBSS for 6 h and CQ (50 μM) for 3 h, photographed by transmission electron microscopy (left). Low-magnification images were on the left, and high-magnification images were on the right in each group. Red arrowheads on the high-magnification images indicated autophagosomes. The numbers of autophagosome per cell were counted, and the difference was analyzed using Prism 5 software (right). The data were presented in the form of mean ± SD, *P < 0.05, **P < 0.01, ***P < 0.001. **e** H4-shRNA cells were seeded onto the coverslips for 24 h, and then transiently transfected with GFP-LC3 for 48 h. The cells were treated with HBSS for 6 h and CQ (50 μM) for 3 h, photographed by fluorescence microscope (top). Scale bar: 10 μm. The average number of autophagosome per cell (nine cells) were quantified manually. The data were presented in the form of mean ± SD, *P < 0.05, **P < 0.01, ***P < 0.001

To clarify the mechanism of TOPK inhibition of autophagy, and in which stage of the autophagy process is involved. OTS964 was used to treat Hs683 and H4 cells, and bafilomycin A1 (Baf A1) was used to block the fusion of autophagosomes with lysosomes, and LC3-II was detected. The results showed that the expression of LC3-II increased with or without the treatment of Baf A1 (Fig. 2a), indicating that inhibition of TOPK activated autophagy initiation without affecting autophagic flux. Then, HBSS was used to activate autophagy and CQ to block the fusion of autophagosomes with lysosomes in TOPK-silencing cell lines, and LC3-II was detected. The results showed that the expression of LC3-II in TOPK-silencing groups was higher than the non-silencing groups with or without the treatment of HBSS or CQ (Fig. 2b, c), indicating that silencing TOPK activated autophagy initiation without affecting autophagic flux. Furthermore, TOPK-silencing H4 cell lines were treated with HBSS or CQ, and the number of autophagosomes was observed under a transmission electron microscope. The results showed that the number of autophagosomes in the TOPK-silencing group was significantly increased compared with the non-silencing group, with or without the treatment of HBSS or CQ (Fig. 2d), indicating that silencing TOPK increased the number of autophagosomes. Then, TOPK-silencing H4 cell lines were transfected with GFP-LC3 and treated with HBSS or CQ, and the number of green dots was observed to reflect autophagosomes. The results showed that the number of green dots was more pronounced in TOPK-silencing groups than in the non-silencing group, with or without the treatment of HBSS or CQ (Fig. 2e), indicating that silencing TOPK promoted the formation of autophagosomes. In summary, TOPK inhibits autophagy initiation in glioma cells.

### TOPK inhibition increases the sensitivity of glioma cells to TMZ through induction of autophagy

**Fig. 3:**
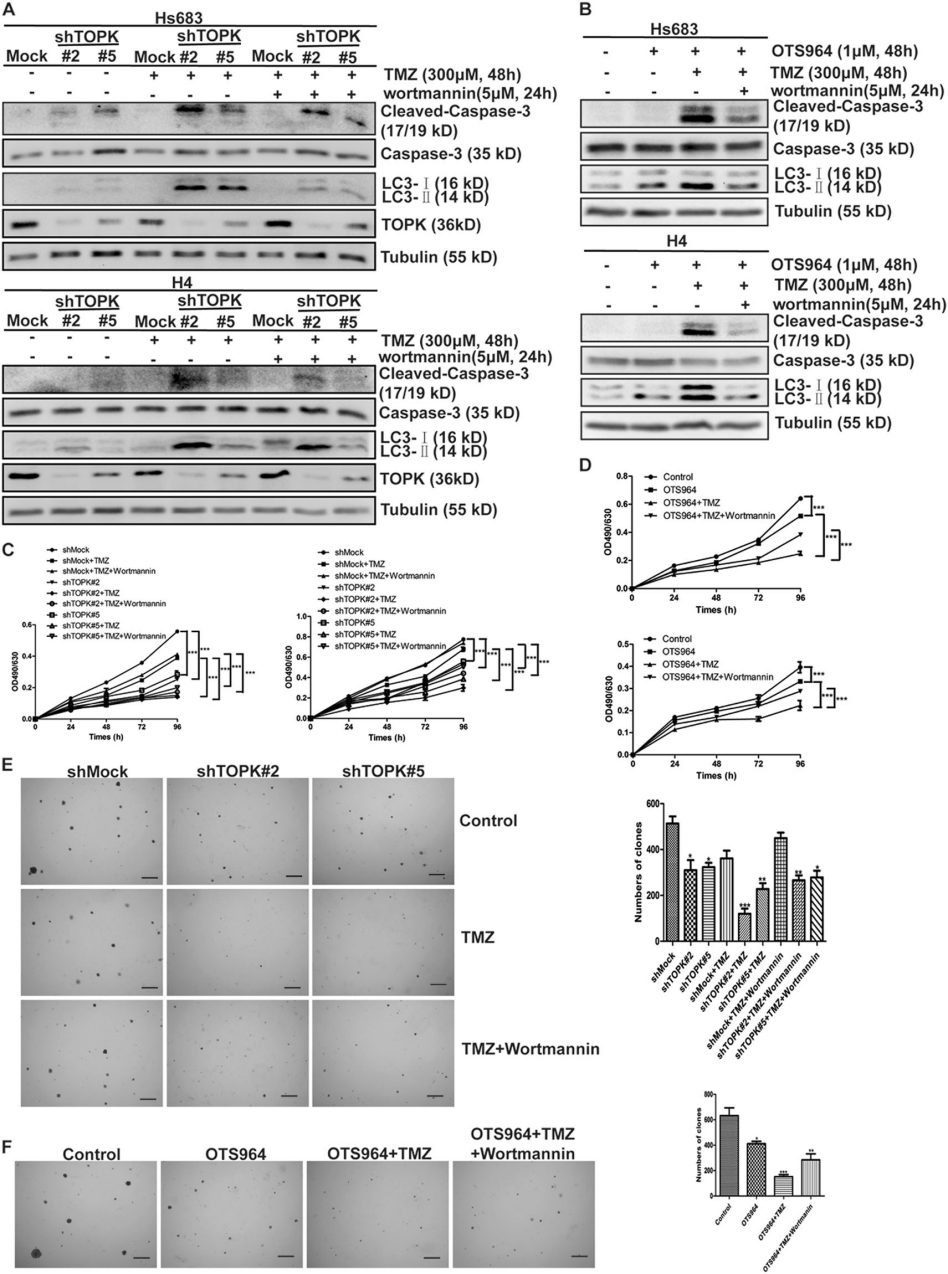
TOPK inhibition increases the sensitivity of glioma cells to TMZ through induction of autophagy. **a** TOPK-silencing-Hs683 (top) or TOPK-silencing-H4 (bottom) cells were treated with TMZ (300 μM, 48 h) and wortmannin (5 μM, 24 h). Samples of whole cell lysates were analyzed using western blots. **b** Hs683 (top), H4 (bottom) cells were treated with OTS964 (1 μM, 48 h), TMZ (300 μM, 48 h), and wortmannin (5 μM, 24 h). Samples of whole cell lysates were analyzed by western blots using indicated antibodies. Data represent the results of triplicate experiments. **c** TOPK-silencing-Hs683 (left) or TOPK-silencing-H4 (right) cells were treated with TMZ and wortmannin in MTT assay. **d** Hs683 (top) or -H4 (bottom) cells were treated with TMZ and wortmannin in MTT assay. **e** TOPK-silencing-H4 cells were treated with TMZ and wortmannin in softagar assay (left). Scale bar: 100 μm. The numbers of clones were analyzed using Prism 5 software (right). The data were presented in the form of mean ± SD, *P < 0.05, **P < 0.01, ***P < 0.001. **f** H4 cells were treated with OTS964, TMZ, and wortmannin in softagar assay (left). Scale bar: 100 μm. The numbers of clones were analyzed using Prism 5 software (right). The data were presented in the form of mean ± SD, *P < 0.05, **P < 0.01, ***P < 0.001

Next, to determine whether the inhibition of autophagy by TOPK affects the sensitivity of glioma cells to TMZ, TMZ was added to TOPK-silencing cell lines, and Cleaved-Caspase-3 and LC3-II were detected. Results showed that administration of TMZ increased the expression of Cleaved-Caspase-3 and LC3-II, and more apparent in TOPK-silencing cells than that of TOPK non-silencing cells (Fig. 3a). This finding indicated that TOPK inhibition increased the sensitivity of glioma cells to TMZ. Further to improve that the increased sensitivity of glioma cells to TMZ after inhibition of TOPK was regulated by autophagy, wortmannin, suppressing the activity of the class III phosphoinositide 3-kinase, was used to inhibit autophagy. Results showed that administration of wortmannin decreased Cleaved-Caspase-3 in all groups, but it was still higher in TOPK-silencing cells than that in TOPK non-silencing cells (Fig. 3a). Similarly, the expression of LC3-II and Cleaved-Caspase-3 increased with the addition of OTS964 and TMZ in Hs683 and H4 cells, as opposed to, the decrease observed in the presence of wortmannin (Fig. 3a). These implied that TOPK played a role in TMZ resistance.

To strengthen these findings, TOPK-silencing cell lines were treated with TMZ and wortmannin in MTT assay and flow cytometry. Results showed that silencing TOPK decreased cell proliferation (Fig. 3c) and increased cell apoptosis (Fig. S1A) with or without the administration of TMZ and wortmannin. Also, TOPK inhibition using OTS964 decreased cell proliferation (Fig. 3d) and increased cell apoptosis (Fig. S1B) of Hs683 or H4 cells with or without the treatment of TMZ and wortmannin. Then, TOPK-silencing H4 cell lines were treated with TMZ and wortmannin in softagar assay. Results showed that silencing TOPK decreased the transformation with or without the administration of TMZ and wortmannin (Fig. 3e). And TOPK inhibition decreased the transformation of H4 cells with or without the treatment of TMZ and wortmannin (Fig. 3f). Taken together, the results indicate that TOPK inhibition increases the sensitivity of glioma cells to TMZ through induction of autophagy.

### TOPK decreases ULK1 activity and stability in glioma cells

**Fig. 4:**
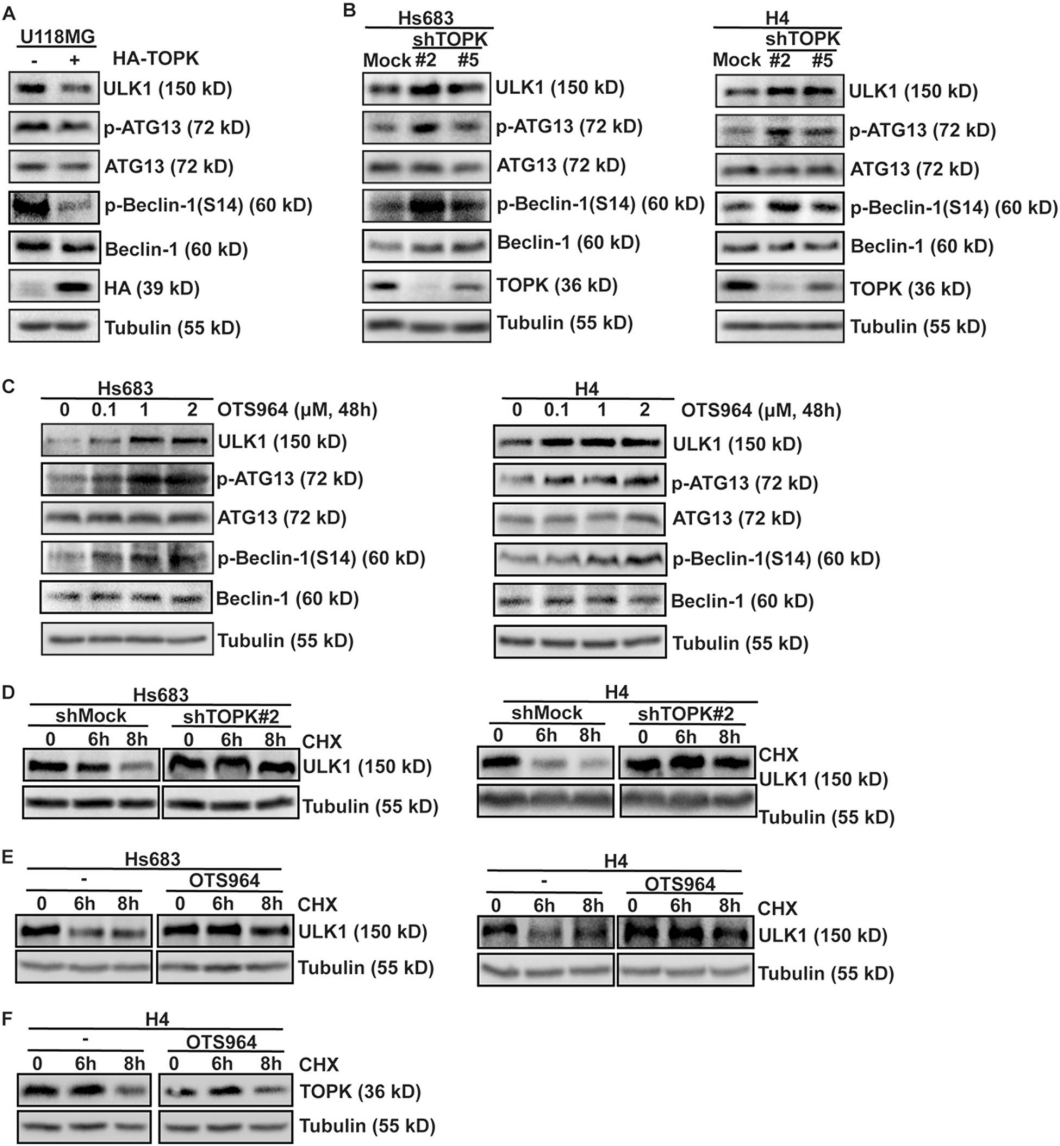
TOPK decreases ULK1 activity in glioma cells. **a** TOPK-overexpression-U118MG cells were harvested at 80% confluence. ULK1, ATG13, p-ATG13, Beclin-1, p-Beclin-1, HA, and Tubulin were analyzed using western blots. **b** TOPK-silencing-Hs683 (left) and TOPK-silencing-H4 (right) cells were harvested at 80% confluence. ULK1, ATG13, p-ATG13, Beclin-1, p-Beclin-1, TOPK, and Tubulin were analyzed using western blots. **c** Hs683 (left) and H4 (right) cells were treated with different concentrations of OTS964 for 48 h, and the samples were resolved by SDS–PAGE and analyzed by western blots using the indicated antibodies. **d** TOPK-silencing-Hs683 (left) and TOPK-silencing-H4 (right) cells were treated with EGF (20 ng/ml) for 15 min followed by the addition of CHX (100 μg/ml) for 6 and 8 h before extraction. ULK1 and Tubulin were analyzed using western blots. **e** Hs683 (left) and -H4 (right) cells were treated with or without OTS964 (1 μM) for 48 h, then cells were exposed to EGF (20 ng/ml) for 15 min followed by the addition of CHX (100 μg/ml) for 6 and 8 h before extraction. ULK1 and Tubulin were analyzed using western blots. **f** H4 cells were treated with or without OTS964 (1 μM) for 48 h, and then cells were exposed to CHX (100 μg/ml) for 6 and 8 h before extraction. TOPK and Tubulin were analyzed using western blots. Data represent the results of triplicate experiments

Next, we continued to explore the mechanism of TOPK-inhibiting autophagy. While ULK1 is required for the initiation of autophagy^32^, we asked whether TOPK could inhibit autophagy by affecting ULK1 activity. For this, we checked the phosphorylation of ATG13 (p-ATG13)^19^ and Beclin-1 (p-Beclin-1)^33^ mediated by ULK1. Results showed that not only the expression of ULK1 but also the phosphorylation levels of ATG13 and Beclin-1 were obviously decreased in TOPK-overexpressing-U118MG cells comparing with that of the control group (Fig. 4a). In contrast, TOPK-silencing cell lines exhibited higher ULK1, p-ATG13, and p-Beclin-1 than that of the control cells (Fig. 4b). Moreover, treatment with OTS964 to selectively inhibit TOPK activity in Hs683 (Fig. 1c) and H4 (Fig. 1d) cells, increased the expression of ULK1 and the levels of p-ATG13 and p-Beclin-1 in a dose-dependent manner (Fig. 4c). The results indicated that TOPK decreases ULK1 activity in glioma cells.

To verify whether TOPK affects the stability of ULK1 based on the changing of ULK1 protein level, TOPK-silencing cell lines were treated with EGF, followed by the addition of CHX before extraction. ULK1 was analyzed. The results showed a longer half-life of ULK1 in TOPK-silencing cells than that of TOPK non-silencing cells (Fig. 4d), indicating an important role of TOPK in the stabilization of ULK1. Furthermore, Hs683 and H4 cells were treated with or without OTS964, and then cells were exposed to EGF, followed by the addition of CHX before extraction, and ULK1 was analyzed. The results showed that the addition of OTS964 extended the half-life of ULK1 in Hs683 and H4 cells (Fig. 4e). However, the addition of OTS964 did not affect on the half-life of TOPK in H4 cells (Fig. 4f). Overall, the data indicate that TOPK decreases ULK1 activity and stability in glioma cells.

### TOPK directly binds with and phosphorylates ULK1 at Ser469, Ser495, and Ser533

**Fig. 5:**
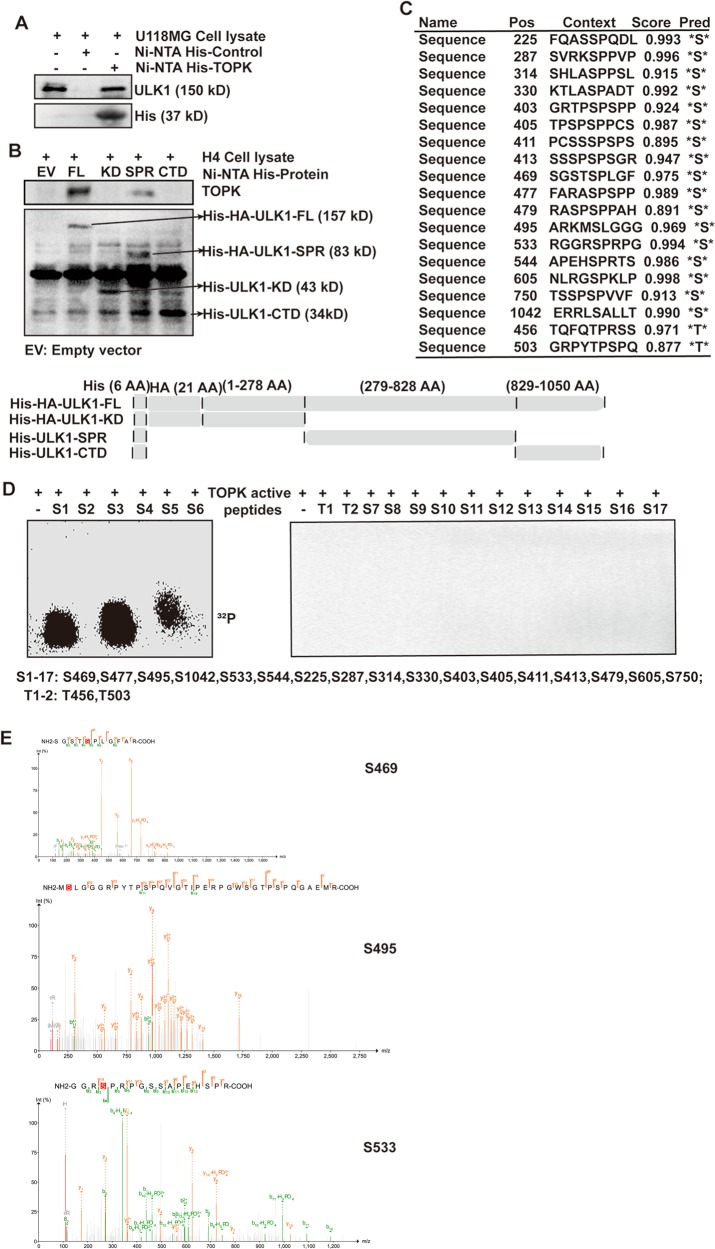
TOPK directly binds with and phosphorylates ULK1 at Ser469, Ser495, and Ser533. **a** TOPK directly bound with endogenous ULK1 of U118MG cells. Purified recombinant Ni-NTA His·Bind Resin-TOPK or Ni-NTA His·Bind Resin was mixed with U118MG cell lysate. Ni-NTA His·Bind Resin was used as a negative control. **b** Endogenous TOPK of H4 cells directly bound with ULK1-SPR. Purified recombinant Ni-NTA His·Bind Resin-ULK1-FL (full length), Ni-NTA His·Bind Resin-ULK1-KD (Kinase Domain), Ni-NTA His·Bind Resin-ULK1-SPR (Serine Proline-Rich), and Ni-NTA His·Bind Resin-ULK1-CTD (C-Terminal Domain) or Ni-NTA His·Bind Resin-EV(empty vector) was mixed with H4 cell lysate. Ni-NTA His·Bind Resin-EV was used as a negative control. Schematic diagram of ULK1 structure and eukaryotic expression construction of each domain was shown in the below. **c** NetPhos2.0 software program was used to predict the potential phosphorylation serine and threonine sites of ULK1. **d** TOPK phosphorylated ULK1 at Ser469, Ser495, and Ser533 in an in vitro kinase assay. TOPK served as an active kinase and ULK1 peptides as substrates. Active TOPK was incubated with each peptide in the presence of [γ-^32^P] ATP, followed by autoradiography. **e** A mass spectrometry (MS) assay was carried out to analyze the phosphorylation modification of ULK1 after an in vitro kinase assay in which TOPK served as an active kinase and His-ULK1-FL protein as substrate

To verify whether TOPK could interact with ULK1, U118MG cell lysate was incubated with Ni-NTA His-TOPK or Ni-NTA His-control in a pull-down assay. The results showed that ULK1 was detected in the Ni-NTA-His-TOPK group (Fig. 5a), indicating that TOPK could bind with ULK1 in vitro. To clarify the specific parts of the combination of TOPK and ULK1, we made three truncated ULK1 mutations, and obtained the full length and three truncated mutations of ULK1 binding with Ni-NTA His·Bind Resin. They were Ni-NTA His-ULK1-FL (full length), Ni-NTA His-ULK1-KD (Kinase Domain), Ni-NTA His-ULK1-SPR (Serine Proline-Rich), and Ni-NTA His-ULK1-CTD (C-Terminal Domain) (Fig. 5b, bottom). Then, H4 cell lysate was incubated with Ni-NTA-His-ULK1-FL/KD/SPR/CTD beads, and TOPK was detected. The results showed that TOPK was detected in the Ni-NTA-His-ULK1-FL and Ni-NTA-His-ULK1-SPR groups (Fig. 5b, top), indicating that TOPK bound to the SPR domain of ULK1.

Then, an in vitro kinase assay was used to verify whether TOPK could directly phosphorylate ULK1 and identify specific phosphorylation sites. First, NetPhos2.0 was used to predict the potential phosphorylation sites of ULK1, and the sites with a high score were selected (Fig. 5c) to synthesize the corresponding peptides. Then, the peptides and [γ-^32^P] ATP as substrates, active TOPK was used as a kinase for an in vitro kinase assay, and autoradiography was used to detect phosphorylation signals. Figure 5d showed that the isotope signal was detected in the peptide containing Ser469, Ser495, and Ser533, suggesting that TOPK phosphorylated ULK1 at Ser469, Ser495, and Ser533. To further confirm that TOPK could phosphorylate the three sites of ULK1 in whole protein, an in vitro kinase assay was carried out using His-ULK1-FL protein as the substrate and identified by MS. As shown in Fig. 5e, MS detected phosphorylated sites of ULK1, including Ser469, Ser495, and Ser533.

The results indicate that TOPK can interact with the SPR domain of ULK1 and phosphorylate ULK1 at Ser469, Ser495, and Ser533.

### The phosphorylation of ULK1 at Ser469, Ser495, and Ser533 inhibits its activity and decreases stability

**Fig. 6:**
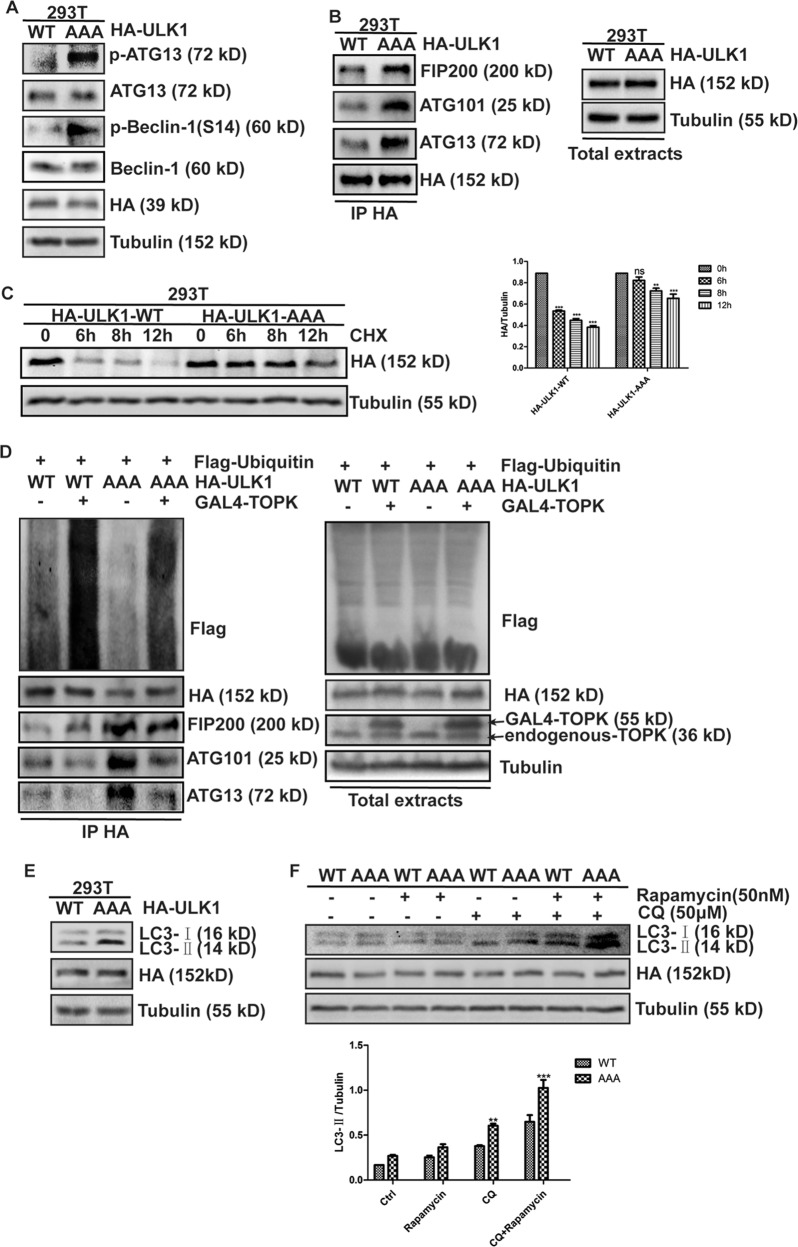
TOPK phosphorylates ULK1 at Ser469, Ser495, and Ser533, inhibiting its activity and reducing its stability. **a** PRK5-HA-ULK1-WT and PRK5-HA-ULK1-Ser469/495/533AAA were transiently transfected into HEK293T cells as indicated. ATG13, p-ATG13, Beclin-1, p-Beclin-1, HA, and Tubulin were analyzed using western blots. **b** PRK5-HA-ULK1-WT and PRK5-HA-ULK1-Ser469/495/533AAA were transiently transfected into HEK293T cells, 48 h later, cells were extracted, immunoprecipitated with anti-HA, and probed with anti-FIP200, anti-ATG101, and anti-ATG13 by western blots. Equal protein loading and transfection efficiency were determined by western blots using the total extracts. **c** PRK5-HA-ULK1-WT and PRK5-HA-ULK1-Ser469/495/533AAA were transiently transfected into HEK293T cells as indicated. Then cells were treated with EGF (20 ng/ml) for 15 min, followed by the addition of CHX (100 μg/ml) for 6, 8, and 12 h before extraction. HA and Tubulin were analyzed using western blots (left). Data of **c** represent the results of triplicate experiments. The densities of HA/Tubulin (right) were determined by Image J. The data were presented in the form of mean ± SD, *P < 0.05, **P < 0.01, ***P < 0.001. **d** HA-ULK1-WT or HA-ULK1-AAA, Flag-ubiquitin, and GAL4-TOPK were cotransfected into HEK293T cells, 48 h later, cells were extracted, immunoprecipitated with anti-HA, and probed with anti-Flag, anti-FIP200, anti-ATG101, and anti-ATG13 by western blots. Equal protein loading and transfection efficiency were determined by western blots using the total extracts. **e** PRK5-HA-ULK1-WT and PRK5-HA-ULK1-Ser469/495/533AAA were transiently transfected into HEK293T cells as indicated. Cells were harvested 48 h later. LC3B, HA, and Tubulin were analyzed using western blots. **f** PRK5-HA-ULK1-WT and PRK5-HA-ULK1-Ser469/495/533AAA were transiently transfected into HEK293T cells as indicated. 24 h later, cells were starved for 24 h, then rapamycin (50 nM) and CQ (50 μM) were added, respectively, for 15 min and 1 h. LC3B, HA, and Tubulin were analyzed using western blots. The densities of LC3-II/Tubulin (Fig. 6**f**, bottom) were determined by Image J. The data were presented in the form of mean ± SD, *P < 0.05, **P < 0.01, ***P < 0.001. Data represent the results of triplicate experiments

To confirm the significance of these three residues in regulating ULK1 activity, a three-site inactivation mutant plasmid of Ser469, Ser495, and Ser533 of ULK1 (ULK1-AAA) was constructed using site-directed mutagenesis, then transfected into HEK293T cells, and p-ATG13 and p-Beclin-1 were detected. Results showed that the levels of phosphorylated ATG13 and Beclin-1 in the ULK1-AAA group were higher than those in the ULK1-WT group (Fig. 6a), suggesting that Ser469, Ser495, and Ser533 site-inactivation mutant of ULK1 enhanced its catalytic capacity. To determine whether ULK1-AAA interacts with other members of its complex to increase their kinase catalytic activity, ULK1-WT or ULK1-AAA was transfected in HEK293T cells, then the cells were lysed to extract total cellular protein, immunoprecipitated with anti-HA antibody, and FIP200, ATG101, and ATG13 were detected. The results showed that in the state of ULK1-AAA, its binding with FIP200, ATG101, and ATG13 increased (Fig. 6b), suggesting that ULK1-AAA enhanced its kinase catalytic activity by affecting its interaction with other members of its complex. The above results indicate that TOPK phosphorylates ULK1 at Ser469, Ser495, and Ser533 to reduce the catalytic activity of ULK1 by attenuating the interaction between ULK1 and other members of the ULK1 complex.

To further explore the mechanism by which TOPK reduces ULK1 stability, and explore whether the change in ULK1 stability is related to its ubiquitination level. First, HEK293T cells were transfected with ULK1-WT or ULK1-AAA, and treated with EGF to activate intracellular TOPK, then treated with CHX to inhibit ribosomes function and to block protein synthesis. HA-tag was detected to observe the stability of HA-ULK1 protein in cells under different treatment conditions. The results showed that the ULK1-WT group was almost completely degraded after 6 h of CHX treatment, while the degradation of ULK1-AAA group was not obvious (Fig. 6c), indicating that the half-life of ULK1 in cells transfected with ULK1-AAA was longer than that of the control group. Then, Flag-Ubiquitin, GAL4-TOPK, HA-ULK1-WT, or HA-ULK1-AAA were co-transfected into HEK293T cells. Then, the cells were lysed to extract total cellular protein, probed with anti-HA antibody, and Flag-tag was detected. The results showed that the ubiquitination level of ULK1 was elevated in the presence of TOPK, while the mutation of ULK1 reduced its ubiquitination level (Fig. 6d), indicating that TOPK reduced the stability of ULK1 by promoting ubiquitination degradation of ULK1. In addition, we observed that the presence of TOPK reduced the binding of ULK1 with FIP200, ATG101, and ATG13, while the mutation of ULK1 increased the binding (Fig. 6d). The above results indicate that TOPK phosphorylates ULK1 at Ser469, Ser495, and Ser533, promoting ubiquitination degradation of ULK1 and reducing its stability.

In order to clarify the effects of Ser469, Ser495, and Ser533 of ULK1 phosphorylated by TOPK on the autophagy, HEK293T cells were transfected with ULK1-WT or ULK1-AAA, and LC3-II was detected. The results showed that the mutation of ULK1 significantly increased the expression of LC3-II (Fig. 6e), indicating that the mutation of ULK1 promoted autophagy. Then, rapamycin was used to inhibit mTOR activity to induce autophagy, and CQ was used to block autophagic flux, and LC3-II was detected. The results showed that the expression of LC3-II in the ULK1-AAA group was significantly increased after treatment with Rapamycin or CQ compared with the ULK1-WT group (Fig. 6f). The results indicate that Ser469, Ser495, and Ser533 of ULK1 phosphorylated by TOPK inhibited the initiation of autophagy.

### Mutation of Ser469, Ser495, and Ser533 of ULK1 induces autophagy initiation and enhances the sensitivity of glioma cells to TMZ

**Fig. 7:**
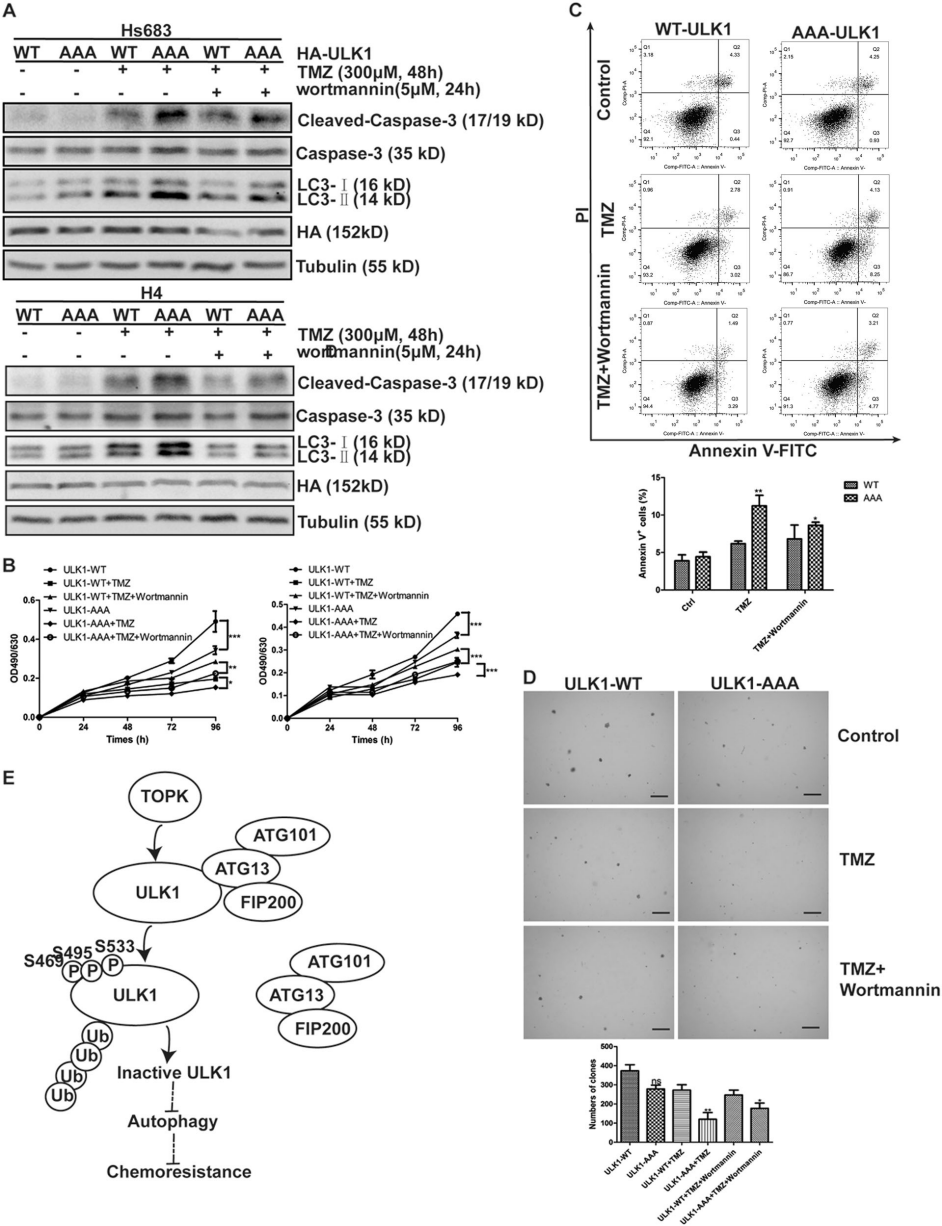
Mutation of Ser469, Ser495, and Ser533 of ULK1 induces autophagy initiation and enhances sensitivity of glioma cells to TMZ. **a** ULK1-WT/AAA-overexpression-Hs683 (top) or -H4 (bottom) cells were treated with TMZ (300 μM, 48 h) and wortmannin (5 μM, 24 h). Samples of whole cell lysates were analyzed using western blots. Data represent the results of triplicate experiments. **b** ULK1-WT/AAA-Hs683 (top) or -H4 (bottom) cells were treated with TMZ and wortmannin in MTT assay. **c** ULK1-WT/AAA-H4 cells were treated with TMZ and wortmannin in flow cytometry (top). The percent of apoptosis cells was analyzed using Prism 5 software (bottom). The data were presented in the form of mean ± SD, *P < 0.05, **P < 0.01, ***P < 0.001. **d** ULK1-WT/AAA-H4 cells were treated with TMZ and wortmannin in softagar assay (top). Scale bar: 100 μm. The numbers of clones were analyzed using Prism 5 software (bottom). The data were presented in the form of mean ± SD, *P < 0.05, **P < 0.01, ***P < 0.001. **e** TOPK phosphorylates ULK1 at Ser469, Ser495, and Ser533, promoting ubiquitination degradation of ULK1, reducing its stability, inhibiting the binding of ULK1 to other members of its complex, and inhibiting the activity of ULK1, therefore inhibiting the initiation of autophagy and promoting the resistance of glioma cells to TMZ

To explore whether Ser469, Ser495, and Ser533 of ULK1 phosphorylated by TOPK affects the sensitivity of glioma cells to TMZ, Hs683 and H4 cells were transfected with ULK1-WT or ULK1-AAA to establish cell lines stably expressing ULK1 wild type and mutant. The stable cell lines were then treated with TMZ and wortmannin, and LC3-II and Cleaved-Caspase-3 were detected. The results showed that the addition of TMZ significantly increased the expression of LC3-II and Cleaved-Caspase-3, and the expression of both in ULK1-AAA cells was significantly higher than that in ULK1-WT cells. Then autophagy was inhibited by wortmannin, the expression of Cleaved-Caspase-3 in each group was decreased, but its expression in ULK1-AAA cells was still significantly higher than that in ULK1-WT cells (Fig. 7a), indicating that the mutation of ULK1 promoted apoptosis.

The ULK1-WT or ULK1-AAA of stable cell lines were then treated with TMZ and wortmannin, and MTT, flow cytometry, and softagar assay were used to test cell proliferation, apoptosis, and clonal growth ability. The results showed that the mutation of ULK1 decreased cell proliferation (Fig. 7b), increased the cell apoptosis (Fig. 7c), and attenuated cell clonal growth ability (Fig. 7d), and it could be reversed by wortmannin. The above results indicate that the mutation of Ser469, Ser495, and Ser533 of ULK1 induce the initiation of autophagy, promoting apoptosis, inhibiting cell proliferation and cell clonal growth ability, and enhancing the sensitivity of glioma cells to TMZ.

In summary, TOPK phosphorylates ULK1 at Ser469, Ser495, and Ser533, promoting ubiquitination degradation of ULK1, reducing its stability, inhibiting the binding of ULK1 to other members of its complex, and inhibiting the activity of ULK1, therefore inhibiting the initiation of autophagy and promoting the resistance of glioma cells to TMZ (Fig. 7e).

## Discussion

Autophagy plays a very important role in tumorigenesis, tumor development, and tumor treatment. Ubiquitination of MAGE-A3/6-TRIM28 degrades AMPKα1, inhibiting autophagy, and promoting tumorigenesis^34^. The metabolic secretions of astrocytes induce autophagy and promote tumor growth^35^. Autophagy can not only enhance the effect of chemotherapy, but also promote chemotherapy resistance. Under metabolic stress, autophagy provides nutrients required for tumor cell survival, which promotes chemoresistance^36^; on the other hand, excessive autophagy leads to “autophagic cell death”, enhancing the effect of chemotherapy^37,38^. Therefore, the regulation of crucial proteins in autophagy can control the activity of autophagy and play a role in cell fate.

GBM is a malignant tumor with poor efficacy and high recurrence. TOPK overexpression promotes the proliferation of glioma-initiating cells (GIC)^39^, proving that TOPK is related to the occurrence of glioma. Our previous studies have shown that TOPK is associated with glioma resistance to TMZ, but the mechanism is unknown. This study found that inhibiting TOPK glioma cells increased the number of autophagic vacuoles, and enhanced the sensitivity of glioma cells to TMZ. Therefore, the combination of TMZ and TOPK inhibitors, such as OTS964 and pantoprazole, is expected to solve the problem of glioma resistance to TMZ.

Studies show that the combination of CQ and TMZ can enhance the sensitivity of glioma to TMZ and improve its therapeutic effect^40^. CQ is a late-stage inhibitor of autophagy, which inhibits autophagic flux by blocking the fusion of autophagosomes and lysosomes, increasing autophagic vacuole accumulation and promoting apoptosis, indicating that changes in sensitivity of glioma to TMZ are associated with the number of autophagic vacuoles. Our study showed that inhibition of TOPK increased autophagic vacuoles, and the number of autophagic vacuoles increased more after CQ treatment, suggesting that TOPK-regulated autophagy occurred in the initial stage of autophagy. Inhibition of TOPK increased the sensitivity of glioma cells to TMZ, but the addition of wortmannin reduced the sensitivity of glioma cells to TMZ. Wortmannin is an inhibitor of PI3K, which inhibits the initial stage of autophagy. Therefore, autophagy inducers, such as rapamycin, can be used to combine with TMZ to solve the problem of glioma resistance to TMZ, but it needs further study.

ULK1 is a crucial protein for autophagy initiation. It can be divided into three domains: kinase domain, SPR domain, and C-terminal domain^41^. Our study showed that TOPK interacted with the SPR domain of ULK1 and phosphorylated Ser469, Ser495, and Ser533 of ULK1, reducing the activity and stability of ULK1, thus inhibiting autophagy, and promoting glioma resistance to TMZ. The phosphorylation of these three sites of ULK1 plays an important role in the resistance of gliomas to TMZ. We hope that the phosphorylation antibodies of these three sites of ULK1 would be applied to the detection of ULK1 activity in the clinic, which is expected to become a diagnostic index for glioma resistance.

Besides our research, another group has proved that active EGFR phosphorylated Beclin-1 to inhibit autophagy in non-small cell lung carcinoma (NSCLC) cells^42^. We suggest that there are some other vital oncokinases, such as JAK2, that can regulate autophagy, and that they engage autophagy at one of the earliest steps of the entire process so as to affect tumor cells growth, proliferation, and chemotherapy resistance in tumors.

## Supplementary information

Figure S1
