# Supplementary material for: TOPK inhibits autophagy by phosphorylating ULK1 and promotes glioma resistance to TMZ
Source: Cell Death Dis. 2019 Aug 5;10(8):583. doi: 10.1038/s41419-019-1805-9 (PMC6680050; doi:10.1038/s41419-019-1805-9)
Supplement: Supplementary file 1 — Figure S1 [file 41419_2019_1805_MOESM1_ESM.docx]

**
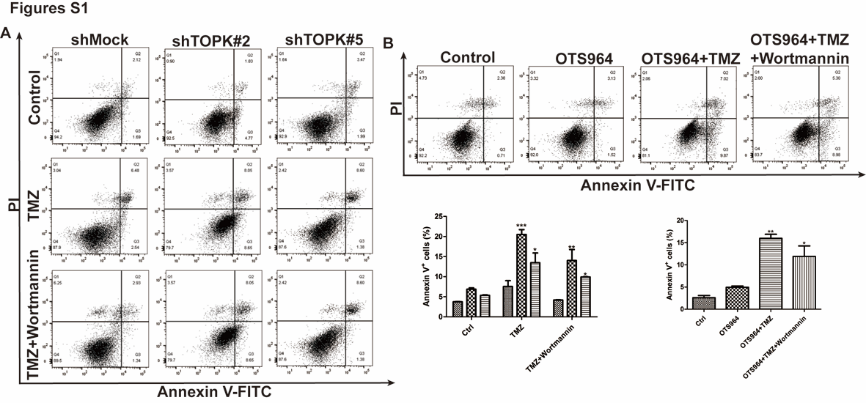
**

**Figure S1. TOPK Inhibition Increases Cell Apoptosis.**

**(A) TOPK-silencing-H4 cells were treated with TMZ and wortmannin in flow cytometry (left). The percent of apoptosis cells was analyzed using Prism 5 software (bottom).** The data were presented in the form of mean ±SD, *P<0.05, **P<0.01, ***P<0.001. (B) **H4 cells were treated with TMZ and wortmannin in flow cytometry (left). The percent of apoptosis cells was analyzed using Prism 5 software (bottom).** The data were presented in the form of mean ±SD, *P<0.05, **P<0.01, ***P<0.001.

**Authors’ contributions**

Lu H, Xiao JJ, Ni XF participated in the experiments. Lu H generated data in almost the whole paper, Xiao JJ prepared panel D in Figure 5, and Ni XF prepared pane C and D in Figure 1. Ke CS, Pan HX provided the clinical specimens. Ji XY, Zhang JM, Xiu RJ, Tian Q, Zou L, Wang F, Ma TF, Yuan P, Liu L offered advice on the manuscript. Lu H, Jia W, Duan QH, Zhu F designed experiments, analyzed data and drafted manuscripts. JS generated the data and prepared panel A, NS generated the immune- histochemistry data and labelled the image, JS assembled the figure.
